# A precision approach to breast cancer treatment based on cell lineage-specific vulnerabilities

**DOI:** 10.18632/oncoscience.538

**Published:** 2021-06-10

**Authors:** Jay S. Desgrosellier

**Affiliations:** ^1^ Department of Pathology, Moores Cancer Center, University of California - San Diego, La Jolla, CA 92093, USA

**Keywords:** breast cancer, stem cells

Breast cancers display significant intra-tumoral heterogeneity posing a major barrier to effective breast cancer treatments [1, 2]. This heterogeneity can be manifested in terms of genetic abnormalities or the presence of distinct cell types bearing similarities to the different epithelial lineages in the normal adult mammary gland including: luminal cells, basal cells, their respective progenitors, and stem cells [3]. The cell lineages present within a given tumor may determine the likelihood of progression. This is best exemplified by sub-populations of tumor-initiating cells present in aggressive breast cancers, some of which resemble adult mammary stem cells [4-9] and thus are termed stem-like. These stem-like cells are enriched in residual tumors after chemotherapy [10] as well as early metastatic lesions [11], suggesting they play a critical role in breast cancer progression. While attempts to treat breast cancers based on genetic mutations have largely been unsuccessful, therapies targeting particular cell lineages, including stem-like cells, are gaining renewed appreciation. Toward this goal, studies have uncovered distinct dependencies among different breast cancer cell types for particular cell death/survival pathways. These recent advances may open the door for new highly personalized approaches to breast cancer therapy.

Our previous studies found that stem-like cells were highly sensitive to cell death induced by p53-upregulated mediator of apoptosis (PUMA) [12], a pro-apoptotic BH3-only member of the Bcl-2 family. These effects were specific to PUMA [12] as the related family member NOXA had no effect on stem-like cells consistent with its role in targeting basal-like breast cancer cells [13]. We further found that driving PUMA expression was sufficient to deplete stem-like cells and reduce metastasis in vivo, revealing its role as an important metastasis suppressor. Our results are consistent with published findings that PUMA-mediated cell death is the preferred response in some normal adult stem cell populations [14]. In an effort to identify a pharmacological means of upregulating PUMA expression, we identified the Src kinase inhibitor dasatinib from an unbiased screen of several clinically-approved targeted therapies. Importantly, our studies showed that dasatinib only induced PUMA expression in stem-like cells, and not other breast cancer cell types. We further showed that PUMA upregulation by dasatinib was capable of depleting stem-like cells and reducing tumor initiation [12]. Taken together, our findings indicated that pharmacologically upregulating PUMA expression with targeted therapies such as the Src inhibitor dasatinib may represent a potential strategy to eliminate stem-like cells and reduce breast cancer metastasis.

**Figure 1:**
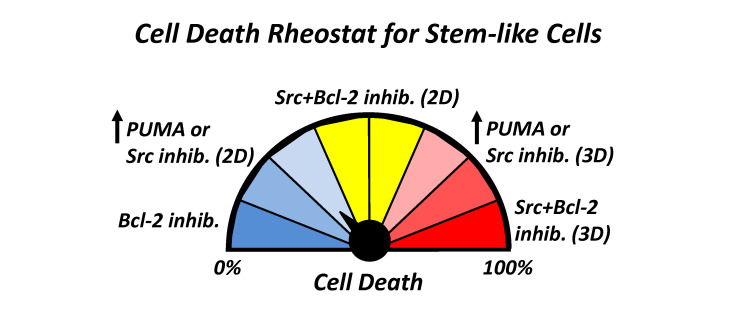
Blocking Bcl-2 enhances cell death due to PUMA in stem-like cells. Response to treatment is also determined by cell context, with enhanced sensitivity in stressful conditions such as suspension (3D) versus adherent (2D) cell culture

While effective at reducing stemness, we also observed that there was little effect of Src inhibition on stem-like cells in adherent conditions, suggesting the presence of innate resistance mechanisms. By examining different pro-survival Bcl-2 family members, we identified the critical PUMA binding protein Bcl-2 [15] as responsible for PUMA resistance. By targeting Bcl-2 with the clinically-approved inhibitor venetoclax, we were able free sequestered PUMA and promote more efficient apoptosis [16], even in adherent cells (Figure 1). While combined Src/Bcl-2 inhibition was a superior therapy to target stem-like cells compared to Src inhibition alone, this treatment was still more effective in anchorage-independent conditions, suggesting that the additional stress enhances response. The ability of tumor cells to grow anchorage-independent tumorspheres is a routinely used in vitro assay to monitor stemness and predictor of metastatic potential. Thus, our findings indicate that this combined therapy may be most effective at targeting disseminating stem-like cells early in the metastatic cascade.

These observations may have important implications for translating our findings to the appropriate clinical setting. Since stem-like tumor cells play a disproportionate role in initiating recurrent and metastatic disease, therapies targeting these cells may best be administered in the adjuvant setting, or post-surgery, to obtain maximum clinical benefit [17, 18]. This is supported by findings that residual tumors after conventional treatment show enrichment for tumor-initiating cells [10]. Consistent with this idea, our prior work showed that driving PUMA expression with the clinically-approved Src inhibitor dasatinib was effective at blocking tumor initiation and metastasis, but not primary tumor growth. This suggested that re-purposing dasatinib to target stem-like breast cancer cells may provide a new use for this drug to prevent new metastases from forming [12]. Our recent study improves upon this work by providing evidence that combining dasatinib with the Bcl-2 selective inhibitor venetoclax produces synergistic cell death in stem-like cells, suggesting it may be even more effective for treating early metastasis. Since it can target cells that aren’t dividing, our combined therapy may also be useful for treating micrometastasis formed by stem-like cells, in contrast with chemotherapy which requires that cells are actively proliferating. Also, since we previously showed that similar stem-like cells reside in both ER- and ER+ primary tumors [12], this suggests that our combination therapy might also be subtype agnostic. Thus, our findings suggest that re-purposing Src and Bcl-2 inhibitors for use in the adjuvant setting may be a more effective use of these drugs, representing a potential therapeutic approach to prevent the emergence of new metastatic disease.

**Figure 2:**
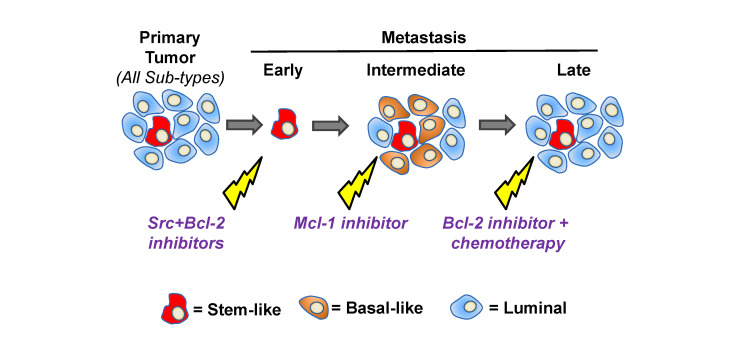
A potential precision therapy approach to target different cancer cell lineages involved in metastatic progression.

While effective as single agents in certain leukemias, Bcl-2 family inhibitors have been less successful in solid tumors [19], suggesting they may work best in combination with other treatments. This may be due to a requirement for induction of pro-apoptotic activators downstream of p53 [20], which is often deficient in breast cancers. Consistent with this idea, we found that PUMA induced by Src inhibition synergizes with venetoclax to eliminate stem-like cells with no effect on luminal or basal-like cell types. While venetoclax [21] was the first Bcl-2 family inhibitor to be clinically-approved [19, 22, 23], drugs targeting other pro-survival family members such as the Mcl-1 selective inhibitor S63845 [24] are currently being investigated in clinical trials. In contrast with our venetoclax results, we also found that S63845 [24] targets basal-like, but not stem-like cells. Overall, our findings suggest that different breast cancer cell lineages uniquely depend on particular cell death/survival pathways. This is supported by publications showing that induced expression of pro-apoptotic BIM by tamoxifen synergizes with venetoclax in ER+ luminal tumors [25], while in a different study, reduced expression of pro-survival Mcl-1 preferentially killed basal-like cells after upregulation of its binding partner NOXA [13]. Mcl-1 inhibitors were also effective against Triple-negative and HER2+ patient-derived xenograft models when combined with chemotherapy [26]. Thus, targeting specific mechanistically linked Bcl-2 family cell death/survival factor pairs may represent a general approach to eliminate certain types of breast cancer cells representing different mammary epithelial lineages within a given tumor (Figure 2). Taken together with our own findings, this may open the door for more individualized therapy for breast cancer by targeting not only stem-like cells, but also other aggressive and potentially compensatory cell types.

Taken together, these recent findings lay the groundwork for a new precision therapy approach for treating breast cancer according to the cell lineages present in a patient’s tumor. Future studies defining the sentinel cell death/survival pathways in additional breast cancer cell types will help identify the most effective personalized treatment for each cancer. Central to this effort will be the elucidation of the most critical pro- or anti-apoptotic proteins to target in each cell type. Observations from our lab and others suggest that different breast cancer cell lineages are dependent on distinct Bcl-2 family members. Additional studies will address the basis for this preference and how this may be altered by differentiation or cell signaling states. This also highlights a need to define the temporal role for each cell type involved in metastasis in order to determine the most effective combination treatments for particular clinical settings, for example to prevent new metastases from occurring post-surgery versus reversing established metastatic disease. To make this a reality, new predictive biomarkers will be needed to detect the particular cell types involved in progression and identify patients who are most at risk of metastasis. These efforts may lead to a new precision therapy approach personalized to the cellular make-up or composition of an individual patient’s tumor, resulting in a major advance in breast cancer treatment. 
